# Professionalism in residents of physical medicine and rehabilitation in Iran

**Published:** 2015-03-15

**Authors:** Tannaz Ahadi, Elaheh Mianehsaz, Gholamreza Raissi, Seyed Alireza Moraveji, Vahid Sharifi

**Affiliations:** 1 Assistant Professor, Department of Physical Medicine and Rehabilitation, Firouzgar Hospital, Iran University of Medical Sciences, Tehran, Iran;; 2 Clinical Research Unit, Shahid Beheshti Hospital, Kashan University of Medical Sciences, Kashan, Iran;; 3 Associate Professor, Department of Physical Medicine and Rehabilitation, Firouzgar Hospital, Iran University of Medical Sciences, Tehran, Iran;; 4 Associate Professor, Department of Community Medicine, Kashan University of Medical Sciences, Kashan, Iran;; 5 Medical Student, Iran University of Medical Sciences, Tehran, Iran.

**Keywords:** professionalism, physical medicine and rehabilitation, ABIM questionnaire

## Abstract

Professionalism is the foundation of trust on which the doctor-patient relationship is built. This study was performed to measure professionalism in Iranian physical medicine and rehabilitation residents as a baseline assessment tool for future studies.

This was a descriptive study. The Persian version of the American Board of Internal Medicine (ABIM) questionnaire was distributed among all the second and third-year residents of the medical state universities of Iran (n=43). Data were summarized as mean (SD), and independent samples t-test was used for comparison of means between genders, and also between the second and third-year residents.

Forty questionnaires were analyzed. The mean (± SD) age of respondents was 29.95 (± 2.37) years. The mean score (SD) for the overall score was 115.15 (± 17.36) out of 150, and the mean score (± SD) for all items was 7.67 (± 1.15) out of 10. The respondents averaged 5.88 (± 1.69) for items forming the ‘excellence’ factor, 7.98 (± 1.48) for items in ‘altruism/respect’ and 8.92 (± 1.26) for items in the ‘honor/integrity’ subscale.

These data may serve as a baseline for future research in this field. The lowest score pertained to excellence, which needs more focus in future studies.

## Introduction

Over the past decades, more global attention has been given to medical professionalism. In Iran, teaching professionalism is an essential part of medical residency training in all programs including physical medicine and rehabilitation (PM&R). Professionalism can be defined as behaviors, goals, or qualities that characterize a profession or a professional person (1) and in medicine it requires physicians to serve patients’ needs and interests above their own (2). In other words, professionalism is the foundation of trust on which the doctor-patient relationship is built. According to the American Board of Internal Medicine (ABIM), professionalism seeks altruism, accountability, excellence, duty, service, honor, integrity, and respect for others (2, 3). 

Traditional medicine in Iran has an inseparable relation with teachings of Avesta (4) comprising codes of medical conduct and characteristics of a good physician. The latter include conscience, compassion, modesty, avoiding abortion and prescription of abortive and lethal drugs and considering the financial situation of patients, and failure to comply with these rules would lead to punishment (5). Later, during the Islamic era, well-known Iranian physicians like Tabari, Avicenna, and Razi dedicated a part of their books to medical conduct (4). In recent years, more research has been conducted on medical ethics and professionalism in medical schools of Iran and other parts of the world. Additionally, several papers have been published focusing on the moral and professional issues in Iranian traditional medicine and historical perspectives (4, 5).

Although professionalism is an element of all physician training and practice, each specialty defines specific professionalism criteria and uses particular questionnaires. ABIM has created a charter for physician professionalism, which defines professionalism for internists (6). Furthermore, the specialty-specific professionalism questionnaire has been reported for several specialties including PM&R (7), emergency medicine (8), and orthopedics (9-11). A widely recognized tool to measure professionalism in medical training environments has been developed by Arnold et al. and is a questionnaire comprising 12 items, each component representing a specific component of professionalism as operationally defined by ABIM (3, 12). Following this study, DeLisa et al. carried out a study applying a revised version of the above-mentioned questionnaire. Theirs was a 12-item questionnaire, which aimed to assess professionalism in PM&R residents of six PM&R residency training programs in the United States (7).

Aramesh et al. assessed the Persian version of the ABIM questionnaire (13). The questionnaire was distributed among residents of 19 fields of specialty (except PM&R) in two major universities of medical sciences in Iran. Results showed that the 15-item Persian version of the modified ABIM questionnaire is a reliable and valid instrument for measuring professionalism (13). Since Aramesh et al. did not assess professionalism in Iranian residents of PM&R, we used this instrument to measure professionalism in Iranian PM&R residents.

## Method

We performed this descriptive study in all 5 medical state universities of the country (Iran, Shahid Beheshti, Shiraz, Tabriz, and Isfahan). In Iran, PM&R is a three-year specialty after internship and there are 7 residency programs educating about 25 residents annually. In the annual PM&R conference a lecture has been presented by Raissi concerning the importance of professionalism in the field of PM&R. Rosters of all medical residents were obtained from the education office of each university. Considering the inadequate presence of first year residents in PM&R departments (since 3 months ago), the questionnaires were distributed among all second and third-year residents (n=43). 

Chief PM&R residents and attending physicians were instructed to explain our goals of the project to all the second and the third-year residents, distribute the questionnaires among them, and once the forms were filled, collect them. Thereafter, the completed questionnaires were sent to the research team by mail. The whole process of distributing, filling out and delivering the questionnaires lasted three months (from December 2012 to February 2013).

In this study, the Persian version of the modified ABIM questionnaire was used. Content validity and internal reliability of this questionnaire was evaluated by Aramesh et al. (13). In the final questionnaire they omitted one question from the original ABIM questionnaire, which was: ‘During this rotation (residency training), I have met individuals whom I consider role models’. The justification was that the concept overlapped with that of another item: ‘During my most recent clinical rotation (residency training), I have encountered individuals who display and promote professional behavior’. Moreover, Aramesh et al. added four new items (12 to 15 as seen in table 1) to the final recommended questionnaire. Thus, their final questionnaire included 15 items in 3 subscales, ‘excellence’, ‘honor/integrity’, and ‘altruism/respect’ (Table 1). Aramesh et al. reported the internal reliability of the scale to be 0.88 based on Cronbach’s alpha, which meets Nunnally’s minimal requirement, and we used this short, reliable and valid questionnaire to measure professionalism in the present study. A comparison of the questions of the original ABIM (2), DeLisa et al. (7) and Aramash et al. (13) are presented in table 2.

Our subjects were also asked whether they had participated in any conferences or workshops on professionalism during their residency-training program. Other questions were about self-training in the subject and their gender, age and the year of study (second or third-year residency).

**Table 1:** Factors and items used for measuring professionalism

*Factor 1: Excellence* 1. During my most recent clinical rotation (residency training), I have encountered individuals who display and promote professional behavior. 2. My residents (resident colleagues) have assisted me in attaining educational material (e.g., journal articles, textbooks) pertaining to my patients. 3. I have observed that my residents (resident colleagues) place the needs of their patients ahead of their own self-interest. 4. I observed that the residents (resident colleagues) I have worked with educate their patients about their illnesses. *Factor 2: Honor/Integrity* 5. I have been instructed to withhold data from a patient’s chart without being given an explanation by my senior resident or attending physician. 6. I have observed my residents (resident colleagues) lie to a patient. 7. The residents (resident colleagues) I have worked with asked me to write orders or fill out forms and sign their names. 8. I have been urged by my residents (resident colleagues) to copy their history and physical examination rather than gather my own information from the patient. *Factor 3: Altruism/Respect* 9. I have observed residents (resident colleagues) referring to patients as ‘hits, gomers, real citizens, walkie-talkies, players, frequent flyers’ or other offensive terms. 10. I have observed residents (resident colleagues) making derogatory statements about other medical/surgical specialty groups or other health care workers. 11. I have observed residents (resident colleagues) scheduling tests or performing procedures at times that are more convenient for themselves than for the patient. 12. I have observed residents (resident colleagues) referring patients to other emergency units or hospitals without a justifiable reason. 13. I have observed residents (resident colleagues) using the materials and equipment of the hospital squanderingly. 14. I have observed residents (resident colleagues) consider their low income and welfare as a reasonable excuse to deliver insufficient services to their patients. 15. I have observed residents (resident colleagues) disclose the private issues of their patients to other residents.

**Table 2:** A comparison of three questionnaires used to measure professionalism

**Items of Questionnaire**	**Arnold et ** **al. (ABIM)**	**DeLisa et ** **al.**	**Aramesh et al. ** **and Current ** **Study**
During my most recent clinical rotation (residency training), I have encountered individuals who display and promote professional behavior.	+	+	+
My residents (resident colleagues) have assisted me in attaining educational material (e.g., journal articles, textbooks) pertaining to my patients.	+	+	+
I have observed that my residents (resident colleagues) place the needs of their patients ahead of their own self-interest.	+	+	+
I have observed that the residents (resident colleagues) I have worked with educate their patients about their illnesses.	+	+	+
During this rotation (residency training), I have met individuals whom I consider role models.	+	_	_
I have observed that the residents I work with actively contribute to the community they serve by participating in community activities outside of their responsibilities at the hospital.	_	+	_
I have been instructed to withhold data from a patient’s chart without being given an explanation by my senior resident or attending physician.	+	+	+
I have observed my residents (resident colleagues) lie to a patient.	+	+	+
The residents (resident colleagues) I have worked with asked me to write orders or fill out forms and sign their names.	+	+	+
I have been urged by my residents (resident colleagues) to copy their history and physical examination rather than gather my own information from the patient.	+	+	+
I have observed residents (resident colleagues) referring to patients as ‘hits, gomers, real citizens, walkie-talkies, players, frequent flyers’ or other offensive terms.	+	_	+
I have observed residents (resident colleagues) making derogatory statements about other medical/surgical specialty groups or other health care workers.	+	+	+
I have observed residents (resident colleagues) scheduling tests or performing procedures at times that are more convenient for themselves than for the patient.	+	+	+
I have observed resident colleagues referring patients to other emergency units or hospitals without a justifiable reason.	_	_	+
I have observed resident colleagues using the materials and equipment of the hospital squanderingly.	_	_	+
I have observed resident colleagues consider their low income and welfare as a reasonable excuse to deliver insufficient services to their patients.	_	_	+
I have observed resident colleagues disclose the private issues of their patients to other residents.	_	_	+

+: Present

-: Absent

**Statistical analysis**

Mean and standard deviation (SD) were measured. Independent samples t-test was used for comparison of means between genders, and also between the second and third-year residents. All statistical analyses were calculated by SPSS software version 21, and *P* values smaller than 0.05 were considered statistically significant.

## Results

Of the 43 distributed questionnaires, 42 were returned (a response rate of 97.6%). However, two sheets were incomplete and were therefore excluded from the study, and a total of 40 sheets were used in the final analysis. The mean (± SD) age of respondents was 29.95 (± 2.37). The mean score (SD) for the overall score was 115.15 (± 17.36) out of a maximum 150. The subjects’ responses to the items forming each factor were also examined. On a scale of 0 to 10 where 10 represents the highest level of professionalism, the mean score (± SD) for all items was 7.67 (± 1.15) out of 10. The respondents averaged 5.88 (± 1.69) for items forming the ‘excellence’ factor, 7.98 (± 1.48) for items in ‘altruism/respect’, and 8.92 (± 1.26) for items in ‘honor/integrity’ factor.

There was no significant difference between the second and third-year, or male and female residents (Table 3).

All the residents had participated in medical ethics, which is a 36-hour course in the undergraduate period. Only four residents had taken part in not only medical conduct classes but also a professionalism workshop or conference, although their responses showed no significant statistical difference compared with others (Table 3). Moreover, five residents had self-training in the field of professionalism, but their responses were not significantly different from the others (Table 3).

**Table 3:** Mean (and SD) of subscale scores stratified by gender, year of study, participation in professionalism workshops, self-training and professionalism of respondents.

Subscale Scores		Excellence Score	Honor Score	Altruism Score	Total Score	*P *Value
Characteristics						
Gender	Male (n = 20)	23.30 ± 6.79	36.55 ± 3.62	55.85 ± 10.96	115.70 ± 17.46	> 0.05
	Female (n = 20)	23.85 ± 6.83	34.85 ± 6.12	55.90 ± 10.07	114.60 ± 17.69	
Year of Study	Year 2 (n = 23)	21.87 ± 6.66	36.04 ± 4.19	57.43 ± 8.82	115.35 ± 14.21	> 0.05
	Year 3 (n = 17)	25.88 ± 6.29	35.00 ± 6.23	53.76 ± 12.16	114.88 ± 21.37	
Professionalism Workshop	Yes (n = 4)	25.25 ± 7.97	36.25 ± 2.06	57.50 ± 4.20	119.00 ± 13.44	> 0.05
	No (n = 36)	23.39 ± 6.68	35.64 ± 5.28	55.69 ± 10.88	114.72 ± 17.84	
Self-Training in Professionalism	Yes (n = 5)	26.6 ± 6.18	37.40 ± 2.40	62.60 ± 3.97	126.60 ± 12.17	> 0.05
	No (n = 35)	23.14 ± 6.77	35.46 ± 5.28	54.91 ± 10.69	113.51 ± 17.50	

## Discussion

Professionalism has had a long history in Iran, and many studies have recently been conducted in this field (13-16). PM&R education is also among topics of interest in the country (17), and PM&R specialists quite often encounter challenging ethical problems in their practice (18). Nevertheless, there has been a shortage of studies on the status of professionalism in Iran during recent years. The present study was conducted on the PM&R residents throughout the country. The response rate in this study was 97.6%, which is higher than that of similar studies, for instance 75% in Arnold et al. (3), and 59% in DeLisa et al. (7). This was due to the cooperation of chief residents and the request of other residents to fill out the questionnaire rather than send them by mail. 

In the present study, the mean score (± SD) for all items was 7.67 (±1.15) out of 10, and this shows a high level of professionalism among the PM&R residents. The same score was 7.7 and 6.12 for DeLisa et al. (7) and Aramesh et al. (13) respectively.

According to our findings the highest score (8.92 ± 1.25) pertained to the ‘honor/integrity’ subscale and the lowest (5.88 ± 1.69) to the ‘excellence’ subscale. The same order of subscales was also reported by DeLisa et al. (7) and Aramesh et al. (13). 

The ‘honor/integrity’ factor shows the point to which the respondents consider their colleagues to be honest and refrain from behaving unprofessionally. Examples of unprofessional conduct are telling lies to patients and encouraging their junior residents to keep data from patients’ charts or to make copies of their history and physical tests (13). In the present study, the highest score pertained to the ‘honor/integrity’ factor, suggesting that the study subjects believed their colleagues were quite honest. 

The ‘altruism/respect’ element signifies the respondents’ view of their colleagues’ respect for their patients, their colleagues, and the rules of the hospital, which prevent them from using resources and tools inefficiently. This subscale also reflects the responders’ views regarding their colleagues’ consideration for patients’ needs and convenience in scheduling tests and procedures (13). 

The ‘excellence’ factor evaluates the responders’ opinions of their colleagues as health service providers who demonstrate and promote professional conduct, help their coworkers, place their patients’ needs above their own, and educate their patients (13). In the present study, the lowest score was reported in the ‘excellence’ subscale signifying that the respondents believed their role models required improvement.

The responses of residents who had participated in professionalism workshops or conferences (n=4) or had self-training in this area (n=5) were not significantly different from those who had not. Nevertheless, this may be due to the small sample size, and further studies are required to investigate the effect of participation in classes, workshops and seminars on the level of professionalism in residents.

## Conclusion

This article is a preliminary study on professionalism in PM&R residents in Iran, and future research is needed to recognize factors affecting health service providers’ level of professionalism. Educational courses can be assessed from this standpoint so that professional training programs and workshops may be added to the PM&R residency curriculum.
